# Clinical Application of an Innovative Multiplex-Fluorescent-Labeled STRs Assay for Prader-Willi Syndrome and Angelman Syndrome

**DOI:** 10.1371/journal.pone.0147824

**Published:** 2016-02-03

**Authors:** Kaihui Zhang, Shu Liu, Bing Feng, Yali Yang, Haiyan Zhang, Rui Dong, Yi Liu, Zhongtao Gai

**Affiliations:** 1 Pediatric Research Institute, Qilu Children’s Hospital of Shandong University, Ji’nan 250022, China; 2 Medical Genetic Center, Guangdong Women and Children's Hospital of Guangzhou Medical University, Guangzhou 511442, China; 3 Pediatric Health Care Institute, Qilu Children’s Hospital of Shandong University, Ji’nan 250022, China; 4 Rehabilitation Center, Qilu Children’s Hospital of Shandong University, Ji’nan 250022, China; National Brain Research Centre, INDIA

## Abstract

Prader-Willi syndrome (PWS) and Angelman syndrome (AS) are two clinically distinct neurodevelopmental disorders caused by absence of paternally or maternally expressed imprinted genes on chr15q11.2-q13.3. Three mechanisms are known to be involved in the pathogenesis: microdeletions, uniparental disomy (UPD) and imprinting defects. Both disorders are difficult to be definitely diagnosed at early age if no available molecular cytogenetic tests. In this study, we identified 5 AS patients with the maternal deletion and 26 PWS patients with paternal deletion on chr15q11-q13 by using an innovative multiplex-fluorescent-labeled short tandem repeats (STRs) assay based on linkage analysis, and validated by the methylation-specific PCR and array comparative genomic hybridization techniques. More interesting, one of these PWS patients was confirmed as maternal uniparental isodisomy by the STR linkage analysis. The phenotypic and genotypic characteristics of these individuals were also presented. Our results indicate that the new linkage analysis is much faster and easier for large-scale screening deletion and uniparental disomy, thus providing a valuable method for early diagnosis of PWS/AS patients, which is critical for genetic diagnosis, management and improvement of prognosis.

## Introduction

Prader-willi syndrome (PWS) and Angelman syndrome (AS) are the first known human genomic imprinting disorders, each with an estimated prevalence of 1/15000-1/25000 in live births. PWS and AS were initially identified by high-resolution chromosome banding, in which patients were found with an interstitial deletion in the same region of chromosome 15q11.2-q13.3 [1]. There is ample evidence that a cluster of genes located in the chromosome region 15q11q13 were expressed from solely paternal or maternal chromosome. It was considered that the deficiency of *UBE3A* gene from the maternal chromosome could lead to AS [1,2]. While the loss of function mutation in SNORD116 snoRNAs could cause the key characteristics of the PWS phenotype [1,3].

PWS patient presents particular manifestations, such as hypotonia, poor suck and feeding difficulties in infancy, then is accompanied by hyperphagia, hypogonadism, hypopigmentation, short stature, cognitive impairments and distinct facial features[4,5]; while AS is characterized by microcephaly, severe intellectual disability, gait ataxia, seizures, an apparent happy demeanor including inappropriate laughter and excitability[6].

PWS is attributed to a deficiency of paternally expressed genes on chromosome 15, usually as the consequence of the paternal deletion (65%-70%), maternal uniparental disomy (mUPD) (20%-30%), mutation or deletion of the imprinting centre(2%-5%) and a translocation of the imprinting center (<1%)[1]. In contrast, AS arises from different molecular defects, including the maternal deletion (70%), paternal uniparental disomy (pUPD) (1%-2%), mutation or deletion of the imprinting centre (2%-4%) and *UBE3A* gene mutations (2%-5%) [5]. Among PWS patients, occurance of mUPD is much higher than that of pUPD in AS patients. The concept of uniparental disomy (UPD) in human was first proposed by Engel in 1980 [6]. The possible mechanisms leading to UPD include monosomy rescue, post-fertilization error, trisomy rescue, gamete complementation, and so on [7].

Both disorders have clinically overlapped features with other diseases during infancy, which makes them difficult to diagnose solely relying on the clinical manifestations. The appearance of hyperphagia and morbid obesity provide useful information for clinical diagnosis of PWS; while the distinctive behavior of apparent happy demeanor and seizures are clinically suspicious of AS. The definite diagnosis of PWS/AS, however, is much dependent on genetic tests. Currently, there are some techniques to identify the disorders, such as G-banding karyotyping, fluorescence in situ hybridization (FISH), methylation-specific PCR (MS-PCR), multiplex ligation-dependent probe amplification (MLPA), short tandem repeat (STR) linkage analysis, DNA sequencing, array comparative genomic hybridization (array CGH) and chromosomal microarray analysis (CMA) [8,9].

In this study, we establish an innovative STR linkage analysis based on multiplx-fluorescent-labeled PCR to detect the AS/PWS patients and then validated by MS-PCR and array CGH. Five AS patients with the maternal deletion and 26 PWS patients with paternal deletion at 15q11-q13 in Chinese population were identified One of the PWS patients was diagnosed as maternal uniparental isodisomy. The clinical characteristics of these individuals were also included.

## Materials and Methods

### Ethics statement

The work was approved by Ethics Committee of Qilu Children’s Hospital of Shandong University and Ethics Committee of Guangdong Women and Children's Hospital of Guangzhou Medical University. The individuals in this manuscript have given written informed consents (as outlined in PLOS consent form) to publish these cases details. The patients’ information was anonymized prior to submission. All the procedures performed in the study were in accordance with the Declaration of Helsinki.

### Patients and samples

This study comprised of 31 patients with PWS /AS (17 male and 14 female, aged from 2 months to 37 year-old) who were diagnosed and recruited from January 2011 to June 2015 in Qilu Children’s Hospital of Shandong University as well as Guangdong Women and children’s Hospital. The diagnosis of PWS and AS was made based on the clinical manifestations and genetic tests. The demographic characteristics and major clinical features of 31 patients were summarized in Table 1.

**Table 1:** Demographics and major clinical features.

Case No.	Clinical diagnosis	Gender	Age	Molecular cytogenetic test results	Major clinical features
1	AS	male	11 months	del(15)(q11.2q13.1)mat	Seizures, happy disposition, speech defect, intellectual disability
2	AS	male	2 months	del(15)(q11.2q13.1)mat	Seizures, delayed psychomotor development
3	AS	female	3 months	del(15)(q11.2q13.1)mat	Seizures, delayed psychomotor development
4	AS	female	12 months	* del(15)(q?11q?13)mat	Seizures, happy disposition, speech defect, severe intellectual disability
5	AS	female	12 months	del(15)(q11.2q13.1)mat	Seizures, happy disposition, speech defect, intellectual disability
6	PWS	female	4 years	UPD(15)mat	Severe intellectual disability, hypotonia in neonate, dysmorphic face
7	PWS	male	2 months	* del(15)(q?11q?13)pat	Hypotonia in neonate, hypopigmentation
8	PWS	female	17 days	* del(15)(q?11q?13)pat	Low-birth weight, hypotonia in neonate, dysmorphic face
9	PWS	male	2 months	del(15)(q11.2q13.1)pat	Hypotonia in neonate, dysmorphic face
10	PWS	male	15 days	* del(15)(q?11q?13)pat	Hypotonia in neonate
11	PWS	female	14 hours	* del(15)(q?11q?13)pat	Hypotonia in neonate, hypoxic ischemic encephalopathy
12	PWS	female	9 years	* del(15)(q?11q?13)pat	Obesity, mental and developmental retardation hypotonia in neonate, short stature, dysmorphic face
13	PWS	male	4 years	* del(15)(q?11q?13)pat	Obesity, hypogonadism, hypopigmentation, severe intellectual disability, hypotonia in neonate, short stature, dysmorphic face
14	PWS	male	9 years	* del(15)(q?11q?13)pat	Obesity, hypogonadism, mental and developmental retardation, hypotonia in neonate, dysmorphic face
15	PWS	male	7 years	* del(15)(q?11q?13)pat	Obesity, hypogonadism, hypopigmentation, mental and developmental retardation, hypotonia in neonate, short stature, dysmorphic face
16	PWS	female	11 years	* del(15)(q?11q?13)pat	Obesity, mental and developmental retardation, hypotonia in neonate, dysmorphic face
17	PWS	female	8 years	* del(15)(q?11q?13)pat	Obesity, severe intellectual disability, hypotonia in neonate, short stature, dysmorphic face
18	PWS	male	6 years	* del(15)(q?11q?13)pat	Obesity, hypogonadism, mental and developmental retardation, hypotonia in neonate, short stature, dysmorphic face
19	PWS	female	14 years	* del(15)(q?11q?13)pat	Obesity, mental and developmental retardation, hypotonia in neonate, dysmorphic face
20	PWS	female	15 years	* del(15)(q?11q?13)pat	Obesity, hypogonadism, mental and developmental retardation, hypotonia in neonate, short stature, dysmorphic face
21	PWS	male	8 years	* del(15)(q?11q?13)pat	Obesity, hypogonadism, mental and developmental retardation, hypotonia in neonate, dysmorphic face
22	PWS	male	7 years	* del(15)(q?11q?13)pat	Obesity, hypogonadism, mental and developmental retardation, hypotonia in neonate, short stature, dysmorphic face
23	PWS	male	15 years	* del(15)(q?11q?13)pat	Obesity, hypogonadism, mental and developmental retardation, hypotonia in neonate, dysmorphic face
24	PWS	male	5 years	* del(15)(q?11q?13)pat	Obesity, hypogonadism, mental and developmental retardation, hypotonia in neonate, short stature, dysmorphic face
25	PWS	female	15 years	del(15)(q11.2q13.1)pat	Obesity, slight intellectual disability, hypotonia in neonate, dysmorphic face
26	PWS	female	37 years	del(15)(q11.2q13.1)pat	Obesity, severe intellectual disability, dysmorphic face
27	PWS	male	6 months	del(15)(q11.2q13.1)pat	Hypotonia, dysmorphic face
28	PWS	male	21 days	del(15)(q11.2q13.1)pat	Low birth weight, hypotonia
29	PWS	female	12 years	del(15)(q11.2q13.1)pat	Obesity, hypogonadism, hypopigmentation, mental and developmental retardation, hypotonia in neonate, short stature, dysmorphic face
30	PWS	male	9 years	del(15)(q11.2q13.1)pat	Obesity, hypogonadism, mental and developmental retardation, hypotonia in neonate, short stature, dysmorphic face
31	PWS	male	4 years	* del(15)(q?11q?13)pat	Obesity, hypogonadism, mental and developmental retardation, hypotonia in neonate, dysmorphic face

The "*"indicated that the accurate deletion region on the chromosome was uncertain because of limited numbers of the microsatellite loci

Peripheral blood samples were collected from the patients and their parents. Genomic DNA was extracted using TIANamp Blood Genomic DNA Purification Kit (Tiangen Biotech, Beijing, China) following the manufacturer’s instructions.

### STR linkage analysis

**Fig 1:**
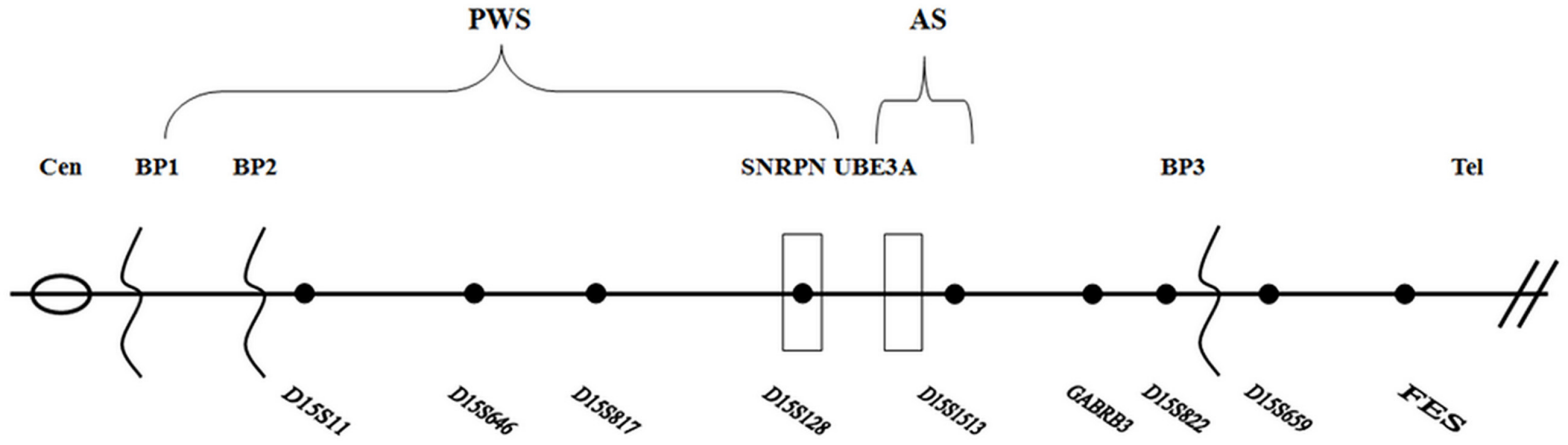
The distribution of the nine STR locus. The black line represents 15q and locations of nine STR loci alleles (D15S11, D15S646, D15S817, D15S1513, GABRB3, D15S822, D15S659 and FES) are shown in dark dots of the line. Seven STR loci alleles are located in the typical PWS/AS deletion region of BP1 to BP3, and the remaining two are located in the distal region near the telomere. Cen: centromere; Tel: telomere; BP: breakpoint.

The STR markers were selected according to their high heterozygosity and location in the typical deletion region (BP1 to BP3) and the adjacent region of PWS and AS. The selected seven STR markers; D15S11, D15S646, D15S817, D15S128, D15S1513, GABRB3, D15S822 were located in the typical PWS and AS deletion region of BP1 to BP3; while additional two loci of D15S659 and FES were in the distal region near telomere. The reference sequence was obtained from the UCSC Genome Browser website (http://genome.ucsc.edu/index.html). The distribution of these loci was shown in Fig 1.

Three multiplex-fluorescent-labeled polymerase chain reactions (PCR A, B and C) were set up with three forward primers labeled different fluoresceins of FAM, JOE and TAMRA in each PCR mixture of A, B and C. The multiplex PCR A was used to identify D15S659 (A1), D15S817 (A2) and D15S1513 (A3). The details of these loci and multiplexes were shown in Tables 2 and 3. The multiplex-fluorescent-labeled PCR A, B and C for all the 9 STR loci was optimized and performed for all the samples from the patients and their parents. The total volume of multiplex-PCR reaction was 50 μL and the reaction condition included: an initial denaturation at 94°C for 5 min, followed by 26 cycles of denaturation at 94°C for 30s, annealing at 61°C for 30s, and extension at 72°C for 30s, plus a final extension at 72°C for 20min. The PCR products were analyzed by capillary electrophoresis on an ABI 3100 sequencer (Applied Biosystems, CA,USA) in the presence of GeneScan 500 LIZ dye Size Standard and formamide (Applied Biosystems, CA, USA). GeneMapper software (Applied Biosystems) was used to provide DNA sizing and quality allele calls.

**Table 2:** Molecular characterization of STRs.

Multiplexes	Microsatellites	Size (bp)	Location	Heterozygosity	Labeled fluorescent
A	A1: D15S659	174–206	15q21.1	0.84	FAM
	A2: D15S817	146–172	15q11.2	0.79	JOE
	A3: D15S1513	232–244	15q12	0.61	TAMRA
B	B1: D15S646	244–256	15q11.2	0.64	FAM
	B2: D15S822	258–306	15q12	0.79	JOE
	B3: FES	142–158	15q26.1	0.68	TAMRA
C	C1: D15S11	243–250	15q11.2	NA	FAM
	C2: D15S128	193–209	15q11.2	0.78	JOE
	C3: GABRB3	144–160	15q12	0.56	TAMRA

**Table 3:** Primers of the MS-PCR and the nine STR loci.

	Primer name	Primer(5’-3’)	Size (bp)
MS-PCR	paternal-specific (unmethylated)	P-F: GTAGGTTGGTGTGTATGTTTAGGT	100bp
		P-R: ACATCAAACATCTCCAACAACCA	
	maternal-specific (methylated)	M-F: TAAATAAGTACGTTTGCGCGGTC	174bp
		M-R: AACCTTACCCGCTCCATCGCG	
STR marker	D15S11	F: AGGCATAACTGCATAGTAAATG	233bp
		R: CAGGCATCCATTTTGAATAGAG	
	D15S646	F: CAGCAATAATGCAAAGGACAGGAG	242bp
		R: AGATGACGGGTTAGTGGGTGCAG	
	D15S817	F: GTATAGAACCGTTCATACTAC	296bp
		R: CTGATGGCATTCAGCCTTA	
	D15S128	F: CATTGCATTTGTATGCAGC	295bp
		R: TCTGTTTTCCTTGCCTGAG	
	D15S1513	F: GGATAAGAAGGATAAAAGTCC	319bp
		R: GAATAAACTTGACATCCTCC	
	GABRB3	F: GCCATTGACTCCAAGAATTCACTCG	418bp
		R: TGATATAACCCTGACTGTGTTTGC	
	D15S822	F: AACTGTATCCAGCATGAATC	340bp
		R: CATAAAGTTAGGTTGATTGAG	
	D15S659	F: TCTACTATATTCAGATTTAGCC	331bp
		R: CATTATTCCTTTTGAGATTCC	
	FES	F: GAAGGTGAAGCCAGTGGGA	338bp
		R: TACTTGGCTACTCGGGAG	

F: forward; R: reverse.

### Methylation-specific PCR analysis

Methylation-specific PCR was performed according to the methods described by Liu et al [10]. The DNA was modified by sodium bisulfite (Takara, Dalian, China), which converts cytosine to uracil except for methylated cytosine that kept unchanged due to being resistant to bisulphate. The imprinting gene *SNRPN* containing a potential imprinting center for a chromosome domain on chr15q11-13, has been indicated that almost all CpG dinucleotides are methylated on the maternal chromosome but unmethylated on the paternal chromosome. The primers for the paternal-specific (unmethylated) and the maternal-specific (methylated) were shown in Table 3. The PCR was carried out in a 25μL reaction solution and the reaction condition included the initial denaturation at 94°C for 5min, followed by 40 cycles of denaturation at 94°C for 30s, annealing at 58°C for 30s, and extension at 72°C for 30s; and a final extension at 72°C for 7min. The PCR amplification products were visualized by a 3% agarose gel.

### Array CGH

Eleven patients (case No: 1,2,3,5,9,25–30) went for the array CGH to detect the chromosomal breakpoints and validate the results of STR linkage analysis. The Agilent Human CGH Microarray 2 x 400K (Agilent, Santa Clara, CA, USA) was utilized following manufacture’s instructions. Data were extracted using Feature Extraction software and analyzed using Agilent Genomic Workbench software (Agilent, Santa Clara, CA, USA). Database such as OMIM (http://www.ncbi.nlm.nih.gov/omim), DECIPHER (http://decipher.sanger.ac.uk/), ISCA (http://dbsearch.clinicalgenome.org/search) and DGV (http://projects.tcag.ca/variation) were used to evaluate the array data and analyze genotype-phenotype correlation.

## Results

### STR linkage analysis

**Fig 2:**
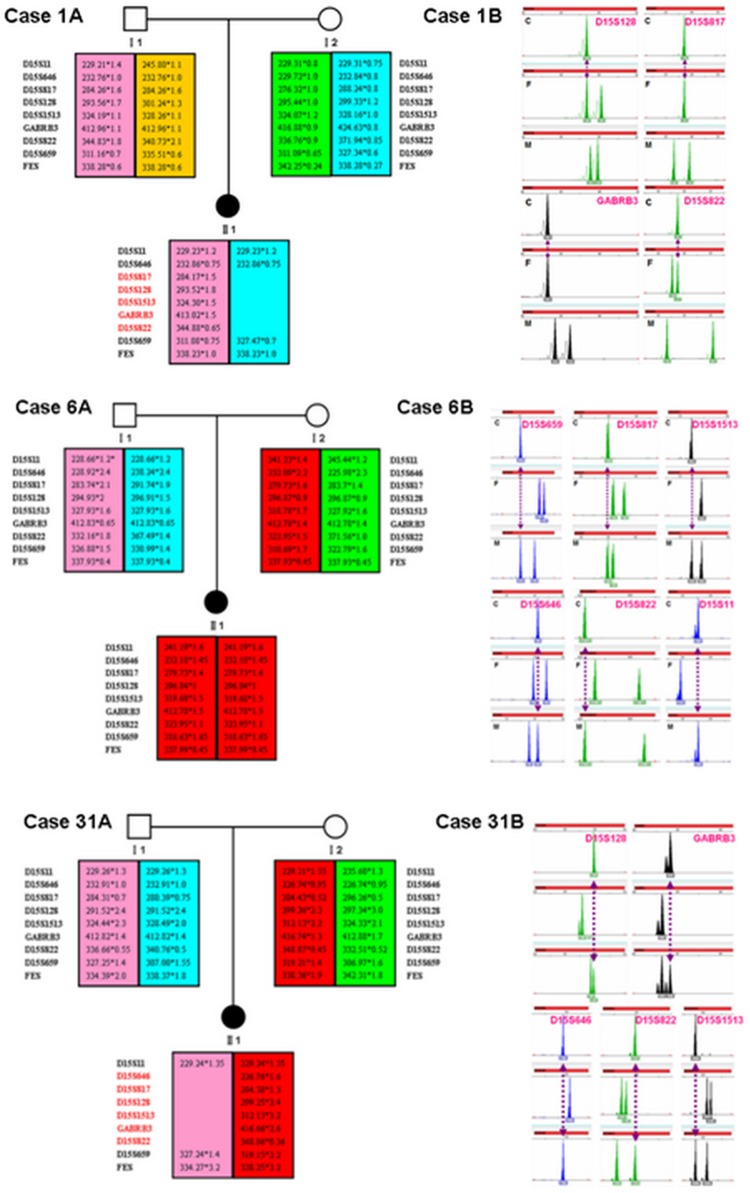
STR linkage analysis results of AS (deletion type) and PWS (deletion type and UPD type). Case 1A/1B: Linkage analysis of family 1 (case 1) showing that the AS patient has the maternal deletion mutation of chromosome 15 fragment (STR D15S817, D15S822, D15S128, GABRB3). Case 6A/6B: Linkage analysis of the family 6 (case 6) showing that the PWS patient inherited two identical chromosome 15 (STR D15S11, D15S646, D15S817, D15S128, D15S1513, GABRB3, D15S822, D15S659 and FES) from her mother and no paternal chromosome 15. Case 31A/31B: Linkage analysis of family 31 (case 31) showing that the PWS patient has the paternal deletion mutation of chromosome 15 fragment (STR D15S1513, D15S646, D15S822, D15S128, GABRB3).

The STR linkage analysis was performed for all the samples from the patients and their parents. Five patients showed the maternal region deletion at the chromosome 15q11-q13 and were confirmed as AS; 26 patients demonstrated the paternal deletion in this region who were diagnosed as PWS. Of them, a 4-year-old girl patient from the family 6 (case 6) displayed two chromosome 15 alleles inherited only from one maternal chromosome 15 that was known as complete uniparental isodisomy of chromosomes 15, thus the patient was eventually identified as PWS with mUPD type. The results of all the patients with AS/PWS tested by STR linkage analysis were listed in Table 1 and S1 Table. The representative results of AS/PWS were from family 1, 6 and 31(case 1, 6 and 31) which were shown in Fig 2.

### Methylation-specific PCR

**Fig 3:**
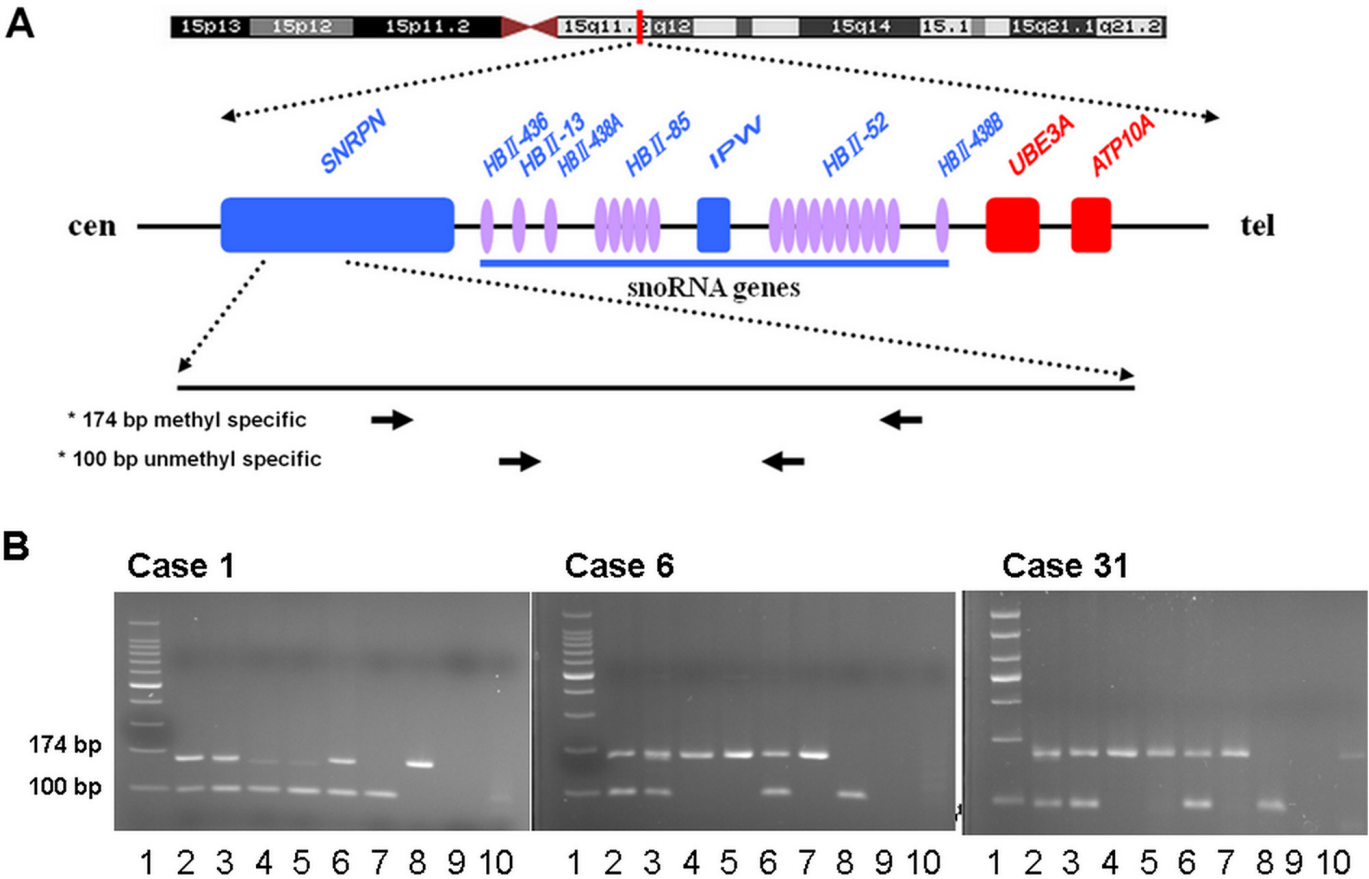
Schematic map of the MS-PCR and MS-PCR analysis for AS/PWS patients. A: Physical map of the SNRPN, UBE3A, and the snoRNA genes. Also shown is the general distribution of the sequence corresponding to the MS-PCR primers for PWS/AS detection. B: MS-PCR analysis of SNRPN gene of AS/PWS patients. MS-PCR products of 174 bp and 100 bp are amplified from methylated and unmethylated alleles of the SNRPN gene locus respectively. Case 1: Lane 1, 100-bp DNA ladder marker; Lane 2, patien’s father; Lane 3, patient’s mother; Lane 4 and 5, case 1 (AS), Lane 6, negative control; Lane 7, AS positive control; Lane 8, PWS positive control; Lane 9, blank control; Lane 10, untreated genomic DNA control. Case 6: Lane 1, 100-bp DNA ladder marker; Lane 2, patient's father; Lane 3, patient's mother; Lane 4 and 5, case 6, PWS, UPD type; Lane 6, negative control; Lane 7, PWS positive control; Lane 8, AS positive control; Lane 9, blank control; Lane 10, untreated genomic DNA control. Case 31: Lane 1, 100-bp DNA ladder marker; Lane 2, patient's father; Lane 3, patient's mother; Lane 4 and 5, case 31, PWS, deletion type; Lane 6, negative control; Lane 7, PWS positive control; Lane 8, AS positive control; Lane 9, blank control; Lane 10, untreated genomic DNA control.

The MS-PCR was carried out to validate the STR results. The products of 174 bp and 100 bp were obtained from methylated and unmethylated alleles of SNRPN gene locus. Five AS patients demonstrated the maternal region deletion at the chromosome 15q11-q13 (with only 100 bp of paternal fragment), while 26 PWS patients showed the paternal deletion in this region (with only 174 bp of maternal fragment). The results were completely in conformity with the STR linkage analysis, thus clearly confirmed that these patients had PWS or AS (Fig 3). However, it could not distinguish whether these PWS/AS patients were caused by deletion, UPD, or other chromosomal abnormalities without the STR linkage analysis.

### Array CGH test

Array CGH test was performed for 11 cases, including 4 AS patients and 7 PWS patients. The results showed that 4 patients with AS had the identical 4.94Mb deletion at 15q11.2—q13.1. However, 7 patients with PWS demonstrated different deletion fragments (S1 Table). These data were well consistent with STR linkage analysis though the deleted fragments were longer than that of STR results, demonstrating the reliability of this innovative STR linkage analysis for identifying PWS and AS.

### Clinical features

Five AS and 26 PWS patients were diagnosed according to the diagnostic criteria described previously [11,12]. In the AS group, all the patients (5/5) had the typical clinical characteristics of AS, such as seizures, delayed psychomotor development, happy disposition, and speech defect. Intellectual disability obviously appeared in over 3 years of age. In the PWS group, all the patients (26/26) had neonatal hypotonia and feeding problems in infancy, but other features presented variable, Of them, 38.46% (10/26) patients demonstrated short stature; 76.92% (20/26) displayed excessive weight gain, hypogonadism, and hyperphagia; 84.62% (22/26) showed dysmorphic features that were distinct especially in child patients. The major clinical features of the 31 patients were listed in Table 1.

## Discussion

Prader-willi syndrome (PWS) and Angelman syndrome (AS) are two clinically distinct neurodevelopmental disorders caused by lack of paternally or maternally expressed imprinted genes at 15q11.2-q13.3, the PWS/AS critical region[1–3]. Three mechanisms are known to be involved in the pathogenesis: microdeletions, UPD and imprinting defects. The genetic tests are indispensable to identify the disorders and there have been some available techniques. For instance, G-banding karyotyping and FISH are traditional methods to detect the large deletions of chromosome. MS-PCR is currently the rapid and convenient test and can accurately identify 99% of PWS and 80% of AS individuals with deletion, UPD, and imprinting defects, but fail to differentiate from one another [2,10,13]. With the specialties of high accuracy, MLPA is known as the “gold standard’ for detection of deletion but has a limitation of inability to differentiate between UPD and imprinting defects [13]. Chromosomal Microarray Analysis (CMA) technology including array CGH with characteristics of high resolution and high throughput has been widely used to detect copy number variants, including UPD in recent years. The sophisticated technology is more propitious to detect isodisomy, but not to heterodisomy. It has been reported that heterodisomy has high occurrence than isodisomy in PWS and AS, even other imprinting diseases [7]. Therefore, STR analysis are required to detect both deletion and UPD status.

STR linkage analysis is a valuable tool to detect the gene location in chromosome with the capability of not only detecting deletion and UPD, but discriminate UPD status [10]. The STR linkage analysis based on direct sequencing could differentiate the molecular defect between typical deletion and UPD, exactly uniparental heterodisomy. However, the STR direct sequencing failed to make a distinction between both types of the deletion and uniparental isodisomy, as no differences between the sequencing results of the deletion and uniparental isodisomy [14]. The STR linkage analysis based on multiplex fluorescent-labeled PCR assay has the advantage over previous sequencing-based STR analysis. The significant and visualized differences of DNA quantity can be observed in the results (S1 Fig). Moreover, the multiplex-fluorescent-labeled STRs assay with only 5 hours, is more simple, rapid, and convenient in comparison with those tedious, time-consuming methods, such as MS-PCR experiment period for 2 days, MLPA for 2 days, STR linkage analysis based on direct sequencing for 3 days and array CGH for 5 days. In addition, the result of the STR linkage analysis is more intuitive. Our data indicated that the multiplex-fluorescent-labeled STRs assay based linkage analysis is an accurate, economic and practical method, not only applicable to identify the PWS/AS, but also diagnose and differentiate almost all imprinting disorders.

UPD is defined as a condition in which both homologous of a pair of chromosomal regions/segments are inherited from only one parent, including isodisomy and heterodisomy. Isodisomy is referred to the inheritance of two copies from a single parental homologue, and heterodisomy is the inheritance of both homologues from one parent [15]. UPD can restore euploidy from a nondisjunction meiotic error to rescue the cell, but it carries two main types of developmental risks: the occurrences of the recessive inheritance diseases and the imprinting diseases. It has been reported that the incidence of UPD was about 1:3500 live births, 2–5% in AS and 25% in PWS [16, 17]. Prader-Willi syndrome is the first known human genomic imprinting disorder, and mUPD of chromosome 15 accounts for 20%-25% in PWS. Complete isodisomy is possibly caused by monosomy rescue, post-fertilization error, or gamete complementation, which there is no reports about the incidence of PWS caused only by isodisomy [15]. Some researches had found that PWS with mUPD was less likely to be hyperphagia and excessive weight gain compared to those with deletion. The subtle phenotypic differences between the deletion and UPD genotypes might also be related to age at diagnosis [18].

In the study, all patients including 5 AS and 26 PWS were diagnosed based on clinical features and genetic analysis. For AS patients, the clinical features of seizures and happy disposition were remarkable in the infant cohort thus the diagnosis was made earlier than that for PWS. The ages of 5 AS patients at diagnosis were from 2 to 12 months. All AS patients (100%) showed seizures which were similar to that reported previously [19]. The apparent happy disposition, such as inappropriate laughter and excitability, was the other unique behavior of the AS patients and noticeable for pediatricians to suspect that child had AS and suggest further genetic tests. Of 5 AS patients, 3 older AS patients at the age of about 1 year old showed happy disposition, whereas other 2 patients aged from 2 to 3 months were too young to have such performance. For PWS, the most significant features are hypotonia in infancy followed by hyperphagia and life-threatening obesity [20], which are overlapped with other diseases during infancy, such as myotonic dystrophy, spinal muscular atrophy and so on[20–23]. As the result, early diagnosis is hard to make and 21 (80.77%) PWS patients were over 4 years old at diagnosis in the cohort of 26 PWS patients. Interestingly, one case (the patient 6) with mUPD was found in 31 PWS/AS (3.6%). She was taken to see a pediatrician because of severe intellectual disability and dysmorphic face. The G-banding karyotyping was initially performed for her showing a polymorphism located in 15ps (not shown) and was highly suspicious of PWS, so the further genetic tests were ordered. After the STR linkage analysis and then validated by MS-PCR and array CGH, she received a diagnosis of PWS with mUPD.

To our knowledge, this is the first time using a multiplex-fluorescent-labeled STRs assay to replace the sequencing-based STR analysis for identification of PWS/AS. Its major advantage over current techniques is the ability to identify near a variety of mutations with three multiplex.The innovative STR analysis provides an accurate, rapid and economic detection method for PWS/AS. It is also the optimal selection to identify the exact molecular defect about genetic imprinting disorders, which is valuable for genetic diagnosis, management of the pediatric patients and improvement of prognosis.

## Supporting Information

S1 Fig Schematic maps of the multiplex-fluorescent-labeled STRs assay for PWS/AS patients with deletion/UPD types. (TIF)

S1 Table Result of genetic analysis for 31 Chinese patients with AS and PWS. (DOC)
