# Supplementary figures and images for: Clinical Application of an Innovative Multiplex-Fluorescent-Labeled STRs Assay for Prader-Willi Syndrome and Angelman Syndrome
Source: PLoS One. 2016 Feb 3;11(2):e0147824. doi: 10.1371/journal.pone.0147824 (PMC4739598; doi:10.1371/journal.pone.0147824)

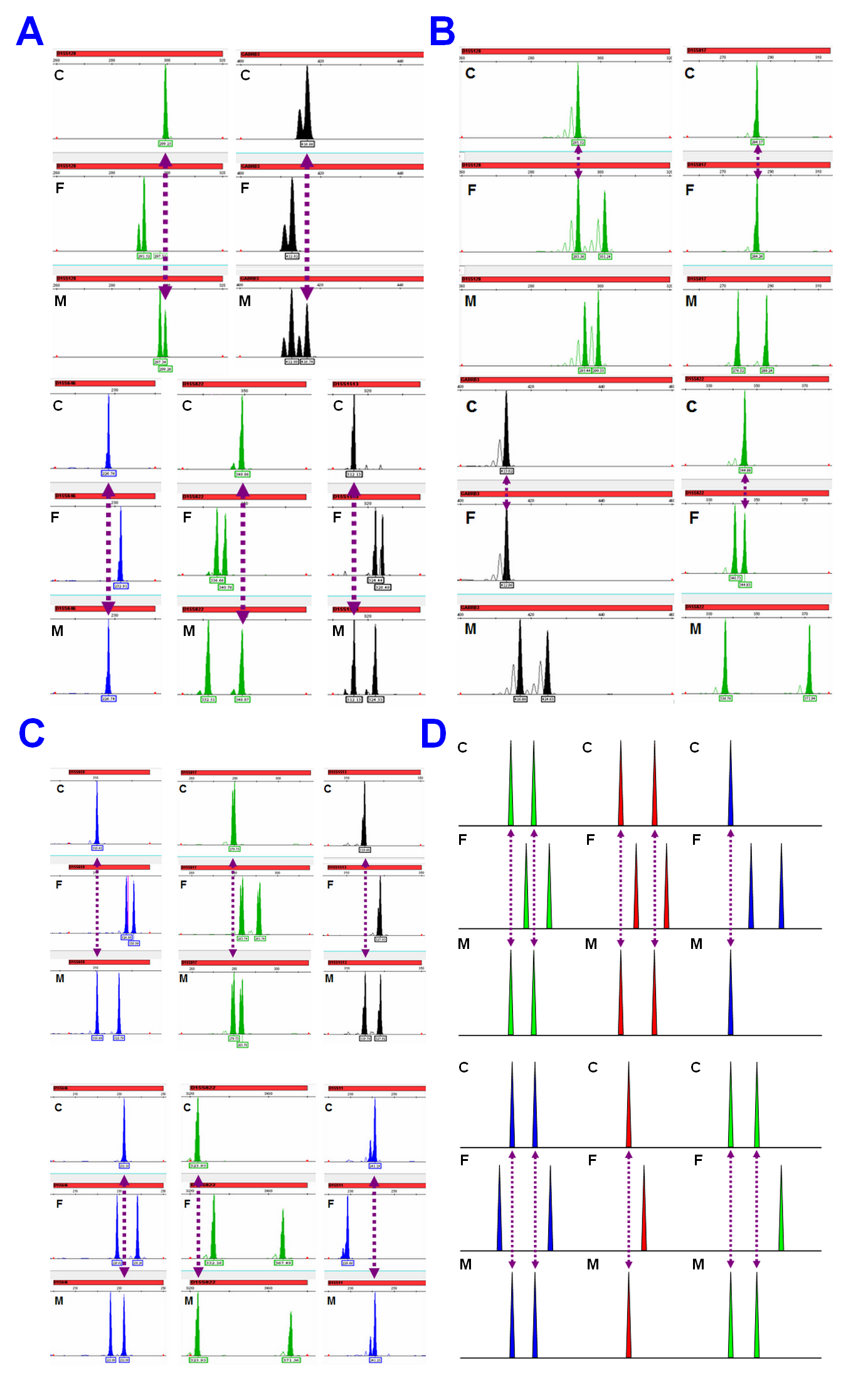

Supplement: S1 Fig — (TIF) [file pone.0147824.s001.tif]
