# Supplementary material for: Clinical Application of an Innovative Multiplex-Fluorescent-Labeled STRs Assay for Prader-Willi Syndrome and Angelman Syndrome
Source: PLoS One. 2016 Feb 3;11(2):e0147824. doi: 10.1371/journal.pone.0147824 (PMC4739598; doi:10.1371/journal.pone.0147824)
Supplement: S1 Table — (DOC) [file pone.0147824.s002.doc]

**Supplemental data**

**S1 Table. Result of genetic analysis for 31 Chinese patients with AS and PWS**

| **Case No.** | **Clinical Diagnosis** | **Affected Locus**  **(Genotyping and linkage analysis)** | **Deletion Size**  **(array CGH)** | **MS-PCR** |
| --- | --- | --- | --- | --- |
| 1 | AS | D15S817-D15S822, 2.81MB | arr15q11.2q13.1(21250794-26199055)×1, 4.94MB | Deletion from maternal chr15 |
| 2 | AS | Not Performed | arr15q11.2q13.1(21250794-26199055)×1, 4.94MB | Deletion from maternal chr15 |
| 3 | AS | Not Performed | arr15q11.2q13.1(21250794-26199055)×1, 4.94MB | Deletion from maternal chr15 |
| 4 | AS | D15S817-D15S822, 2.81MB | Not Performed | Deletion from maternal chr15 |
| 5 | AS | Not Performed | arr15q11.2q13.1(21250794-26199055)×1, 4.94MB | Deletion from maternal chr15 |
| 6 | PWS | D15S11-FES, 69.80MB | Not Performed | UPD of chr15 |
| 7 | PWS | D15S1513-D15S822, 1.24MB | Not Performed | Deletion from paternal chr15 |
| 8 | PWS | D15S11-GABRB3, 2.65MB | Not Performed | Deletion from paternal chr15 |
| 9 | PWS | D15S817-D15S1513, 1.58MB | arr15q11.2q13.1(21250794-26199055)×1, 4.94MB | Deletion from paternal chr15 |
| 10 | PWS | D15S11-D15S822, 3.34MB | Not Performed | Deletion from paternal chr15 |
| 11 | PWS | D15S11-D15S822, 3.34MB | Not Performed | Deletion from paternal chr15 |
| 12 | PWS | D15S11-D15S822, 3.34MB | Not Performed | Deletion from paternal chr15 |
| 13 | PWS | D15S11-D15S822, 3.34MB | Not Performed | Deletion from paternal chr15 |
| 14 | PWS | D15S646-D15S822, 3.07MB | Not Performed | Deletion from paternal chr15 |
| 15 | PWS | D15S11-D15S822, 3.34MB | Not Performed | Deletion from paternal chr15 |
| 16 | PWS | D15S646-D15S822, 3.07MB | Not Performed | Deletion from paternal chr15 |
| 17 | PWS | D15S646-D15S822, 3.07MB | Not Performed | Deletion from paternal chr15 |
| 18 | PWS | D15S11-D15S822, 3.34MB | Not Performed | Deletion from paternal chr15 |
| 19 | PWS | D15S11-GABRB3, 2.65MB | Not Performed | Deletion from paternal chr15 |
| 20 | PWS | D15S817-D15S822, 2.81MB | Not Performed | Deletion from paternal chr15 |
| 21 | PWS | D15S11-D15S822, 3.34MB | Not Performed | Deletion from paternal chr15 |
| 22 | PWS | D15S1513-D15S822, 1.24MB | Not Performed | Deletion from paternal chr15 |
| 23 | PWS | D15S1513-D15S822, 1.24MB | Not Performed | Deletion from paternal chr15 |
| 24 | PWS | D15S11-D15S822, 3.34MB | Not Performed | Deletion from paternal chr15 |
| 25 | PWS | D15S1513-D15S822, 1.24MB | arr15q11.2q13.1(21250794-26199055)×1, 4.94MB | Deletion from paternal chr15 |
| 26 | PWS | D15S646-D15S822, 3.07MB | arr15q11.2q13.1(21290455-26199055)×1, 4.91MB | Deletion from paternal chr15 |
| 27 | PWS | D15S11-D15S822, 3.34MB | arr15q11.2q13.1(18741716-26199055)×1, 7.46MB | Deletion from paternal chr15 |
| 28 | PWS | D15S817-D15S822, 2.81MB | arr15q11.2q13.1(20335887-26199055)×1, 5.86MB | Deletion from paternal chr15 |
| 29 | PWS | D15S128-GABRB3, 1.60MB | arr15q11.2q13.1(21250794-26199055)×1, 4.94MB | Deletion from paternal chr15 |
| 30 | PWS | D15S11-D15S822, 3.34MB | arr15q11.2q13.1(21364382-26199055)×1, 4.83MB | Deletion from paternal chr15 |
| 31 | PWS | D15S646-D15S822, 3.07MB | Not Performed | Deletion from paternal chr15 |

**S1 Fig. Schematic maps of the multiplex-fluorescent-labeled** **STRs assay for PWS/AS patients with deletion/UPD types.**

A: Linkage analysis of case 31 family shows that the patient has the paternal deletion at chromosome 15q, and the result indicates deletion type of PWS (S1 Fig. A).

B: Linkage analysis of case 1 family shows that the patient has the maternal deletion at chromosome 15q, and the result indicates deletion type of AS (S1 Fig. B).

C: Linkage analysis of case 6 family shows that the patient has only one homozygous peak signal detected in every STR locus including the two additional control loci of D15S659 and FES which are not included in the maximal deletion region of PWS/AS; but for the mother, almost all the STR loci are heterozygous including D15S659. These results indicate maternal uniparental isodisomy type of PWS that the patient inherited two identical chromosome 15 from his mother and no paternal chromosome 15(S1 Fig. C).

D: Schema graphs of linkage analysis for uniparental heterodisomy are presented (as no patient of uniparental heterodisomy has been found so far,). The simulated results show that in the PWS patient, the genotype of the STR loci at 15q are identical to the mother, indicating maternal uniparental heterodisomy type of PWS (S1 Fig. D).
